# MiR-1224 Acts as a Prognostic Biomarker and Inhibits the Progression of Gastric Cancer by Targeting SATB1

**DOI:** 10.3389/fonc.2021.748896

**Published:** 2021-09-17

**Authors:** Guo-Dong Han, Yuan Sun, Hong-Xia Hui, Ming-Yue Tao, Yang-Qing Liu, Jing Zhu

**Affiliations:** ^1^Department of Orthopedics, The Affiliated Huaian No.1 People’s Hospital of Nanjing Medical University, Huai’an, China; ^2^Department of Medical Oncology, The Affiliated Huaian No.1 People’s Hospital of Nanjing Medical University, Huai’an, China

**Keywords:** miR-1224, metastasis, SATB1, Wnt/β-catenin, gastric cancer, prognosis

## Abstract

**Objective:**

MiR-1224 has been reported to exhibit abnormal expression in several tumors. However, the expressing pattern and roles of miR-1224 in gastric cancer (GC) remain unclear. Our current research aimed to explore the potential involvement of miR-1224 in the GC progression.

**Materials and Methods:**

The expression of miR-1224 was examined in tissue samples of 128 GC patients and cell lines by RT-PCR. Besides, the associations of miR-1224 expressions with clinicopathologic features and prognosis of GC patients were analyzed. Then, the possible influences of miR-1224 on cell proliferation and cell migration were determined. Afterward, the molecular target of miR-1224 was identified using bioinformatics assays and confirmed experimentally. Finally, RT-PCR and Western blot assays were performed to investigate the effect of the abnormal miR-1224 expression on the EMT and Wnt/β-catenin pathway.

**Results:**

miR-1224 was lowly expressed in the GC specimens and cell lines due to T classification and TNM stage. Survival assays demonstrated that GC patients with low expressions of miR-1224 possessed poor overall survivals. Moreover, *in vitro* and *in vivo* assays revealed that the overexpression of miR-1224 inhibited cell proliferation, migration, and invasion in GC cells. SATB homeobox 1 (SATB1) was verified as a direct target of miR-1224 in GC. Furthermore, β-catenin and c-myc were significantly inhibited in miR-1224-overexpression cells.

**Conclusions:**

Our findings highlight the potential of miR-1224 as a therapeutic target and novel biomarker for GC patients

## Introduction

Gastric cancer (GC) is a malignant tumor originating from the glandular epithelium of the gastric mucosa (1, 2). It is estimated that approximately 1 million new cases of GC are diagnosed per year worldwide (3). The mainstream therapeutic regimen of GC consists of surgical operation and traditional radiations with extra chemotherapy (4, 5). The survival rate in five years for GC has not improved significantly over the past several decades despite the improvements in surgery, radiotherapy, and chemotherapy (6–8). Cancer metastasis and local tumor recurrence have been major reasons for death among GC patients (9). To date, the molecular mechanisms of metastasis and local tumor recurrence has not been unveiled, though increasing efforts have been made to solve it (10). Therefore, investigating the molecular events underlying GC progression would contribute to the development of novel strategies for the clinical management of patients with this tumor.

MicroRNA (miRNA) is small noncoding RNAs of around 22 nucleotides and can mediate mRNA degradation by binding the 3’UTR of target mRNAs (11, 12). They are involved in a wide range of biological processes, including cellular development, differentiation/dedifferentiation, programmed cell death, and cell proliferation (13–15). MiRNAs can act as anti-oncogenes or tumor promoters during the progression and migration of various cancers, including GC, making them very attractive for the detection and prognosis assessment of cancers (16–20). Dysregulated miRNAs expression has been observed in various human malignancies through oligonucleotide miRNA microarray and RT-PCR technologies; some miRNAs have been identified as prognostic markers for some cancers, including GC (21–23). However, there is little research on the biological function of many miRNAs in GC.

MiR-1224, located at 3q27.1, is a recently identified miRNA that has been reported to play an essential role in some diseases, such as cute liver failure, Parkinson’s disease, and cerebral ischemia (24–26). Although some reports have revealed that miR-1224 was abnormally expressed in several tumors, its clinical significance and biological function in tumors remain unclear (27–29). Moreover, the expression pattern and clinical significance of miR-1224 have not been investigated, as well as its potential biological function in GC. Our study demonstrated that miR-1224 served as an anti-oncogenic miRNA in GC progression and could be regarded as a novel biomarker and an efficient therapeutic target in the clinical treatments of GC.

## Materials and Methods

### Patients and Tissue Samples

Human GC specimens and adjacent non-tumor tissue samples were obtained between October 2014 and March 2016 from surgical specimens of patients with GC undergoing surgery at The Affiliated Huaian No.1 People’s Hospital of Nanjing Medical University. The samples were snap−frozen in liquid nitrogen and stored at -80˚C for the next experiments. We obtained written informed consent from all of these patients. This study was approved by the Research Ethics Committee of The Affiliated Huaian No.1 People’s Hospital of Nanjing Medical University.

### Cell Lines and Cell Transfection

Human GC cells, including BGC-823(CBP60477), HGC-27(CBP60480), MKN-45(CBP60432), AGS(CBP60476), SGC-7901(CBP60500), HGC-27(CBP60480) and immortalized normal gastric epithelial cell line GES-1(CBP60512), were grown in RPMI 1640 medium (#2381, Haihu Technology, Haidian, Beijing, China) (with 10% fatal bovine serum(FBS) and 1% antibiotics) and obtained from Nanjing COBIOER Biotechnology Co., Ltd. (Nanjing, Jiangsu, China). Regarding cell transfection, LipoFectMax reagent kits (HYY Medicine, Guangzhou, Guangdong, China) were adopted to transfect miR-1224 mimics (MOI 0.01), miR-1224 inhibitors (MOI 0.01), negative control (NC) inhibitors (MOI 0.01), or negative control (NC) mimics (MOI 0.01) into SGC-25 and SGC-7901 cells. For the analysis of viability 200 cells per concentration weach concentration used were scored. The miRNA mimics and inhibitors used in this study were synthesized by BioTend Biotechnology Co., Ltd. (Huangpu, Shanghai, China).

### Real-Time PCR Assays

Total RNA was extracted from specimens and cells utilizing the TRIzol reagent (PuliKe Biotech, Shapingba, Chongqing, China). Subsequently, qRT-PCR analysis for mRNA (SATB homeobox 1 (SATB1), c-myc, cyclin D1, β-catenin) and miR-1224 examination (Primers were summarized in **Table 1**) were conducted using Cells Direct one step qRT-PCR kit (Thermo Fisher, Pudong, Shanghai, China) and RiBoBio miDETECT miRNA qRT-PCR Starter kit (RiBoBio, Guangzhou, Guangdong, China), respectively, based on an ABI QuantStudio Dx real-time PCR system (Daan Gene, Guangzhou, Guangdong, China) (30). Data were normalized to GAPDH or U6 expression and calculated with the 2^−△△Ct^ methods.

**Table 1:** Primer sequences used for quantitative real-time PCR.

Gene	Sequence
miR-1224	F: 5’-GCCGAGGTGAGGACTCGGG-3’
	R: 5’-TGGTGTCGTGGAGTCG-3’
SATB1	F: GATCATTTGAACGAGGCAACTCA
	R: TGGACCCTTCGGATCACTCA
GAPDH	F: 5’- ACAACTTTGGTATCGTGGAAGG-3’
	R: 5’-GCCATCACGCCACAGTTTC-3’
U6	F: 5’CTCGCTTCGGCAGCACA-3’
	R: 5’AACGCTTCACGAATTTGCGT-3’

### Cell Counting Kit-8 Assays

CCK-8 assays kits were obtained from ExcellBio Technology Co., Ltd. (Taicang, Jiangsu, China). Briefly, a total of 5 × 10^3^ SGC-25 or SGC-7901 cells (per well) were placed into 96-well plates after the transfection of miR-1224 mimics for 24 h. Afterward, the CCK-8 reagent (10 μl) was added at the indicated time. Sequentially, a DNM-9606 microplate reader instrument (PerLong Medical, Shunyi, Beijing, China) was employed to detect the OD value at the absorbance of 450 nm.

### EdU Assays

An EdU Apollo Imaging kit (RiboBio, Guangzhou, Guangdong, China) was employed to conduct the EdU assays (31). Specifically, the miR-1224 mimics or negative control (NC) mimics-transfected SGC-25 or SGC-7901 cells (about 4000 cells per well) were planted into 48-well plates. Twenty-four hours later, EdU solution (50 μM final concentration) was added to each well. The treated cells were maintained for an additional 2 hours and added with DAPI solution (32). Finally, the cells were fixed with formaldehyde (4%) and observed using fluorescence microscopy.

### Colony Formation Assays

BGC-823 and SGC-7901 cells transfected with miR-1224 mimics or NC mimics were placed into 6-well plates (500 cells per well) and maintained in RPMI-1640 medium (10% FBS) for about 2 weeks. Subsequently, PBS was used to wash the cell colonies, and crystal violet solution (0.2%; SNOTA Biotech, Shenyang, Liaoning, China) was utilized to stain the colonies. Finally, the stained colonies were counted by a microscope (YDF-90; YuanRen, Pudong, Shanghai, China).

### Western Blot Assays

First, protein lysates, which were extracted from corresponding miRNA mimics-transfected cells using RIPA buffer, were separated by 8-12% SDS-PAGE. Then, the isolated proteins were transferred to Millipore polyvinylidene fluoride membranes. Thereafter, the primary antibodies targeting proteins were prepared and incubated with the membranes. Next, the examination of these proteins was performed using an ECL assay kit (TellGen, Pudong, Shanghai, China) after the corresponding secondary antibodies were probed with the membranes. The primary antibodies targeting Vimentin (1: 1000, ab92547, Cambridge, UK), N-cadherin (1:500, BD Biosciences, 610,921), β-Catenin(1: 1000, ab68183, Abcam, Shenzhen, China), cyclin D1(1: 1000, ab16663, Abcam, Shenzhen, China) and c-myc(1: 1000, ab32072, Abcam, Shenzhen, China) and GAPDH(1: 1000, ab8245, Abcam, Shenzhen, China) for this experiment were all purchased from NeoBioscience Biotechnology Co., Ltd. (Shenzhen, Guangdong, China).

### Wound Healing Assays

Collected cells were planted into 6-well plates and subsequently grown to 70-80% confluence, followed by transfecting miR-1224 mimics or NC mimics. After culturing for 24 h, the monolayer GC cells were scraped by a sterile 200 µl pipette tip (Xinlong Biology, Xuhui, Shanghai, China). The cells were washed, and pictures of the wounded areas at 0 h and 48 h were taken using a microscope (YDF-90; YuanRen, Pudong, Shanghai, China).

### Transwell Invasion Assay

The invasiveness of cells after treatment was conducted using BD Biosciences 8μm-pore-size Invasion Chambers (MingYang Biotech, Chaoyang, Beijing, China) pre-treated with Matrigel. Cells (1 × 10^4^) in RPMI-1640 medium were placed into the upper chamber while 700 μl 20% FBS was added into the lower chamber. Then, cells invading through the insert membranes were dyed with crystal violet (0.2%; SNOTA Biotech, Shenyang, Liaoning, China). The images were taken using a microscope (YDF-90; YuanRen, Pudong, Shanghai, China).

### Luciferase Reporter Assays

TargetScan was adopted to predict the putative target genes of miR-1224. Luciferase reporter assays were performed following the instructions. The 3’UTR sequence of special AT-rich binding protein 1 (SATB1) predicted to interact with miR-1224 or the mutated sequence was synthesized by Gema Technology (Pudong, Shanghai, China) and then inserted into pGL3-promoter Luciferase vector. Besides, successful clones were determined with sequencing technology. The 293T cells were seeded into 6-well plates for 1 day and co-transfected with 20 ng of the above-synthesized vectors and 20 nM of miR-1224 mimics or control mimics applying Lipofectamine 2000 (Invitrogen, Beijing, Haidian, China). Finally, the luciferase activity 48 hours after transfection was determined using the dual-luciferase reporter assay system (Promega, Hangzhou, Zhejiang, China).

### Tumorigenicity

In nude mice of Balb/c (male, 4-week-old) (n = 6), the tumor growth of SGC7901 cells *in vivo* was explored. About 5 × 10^6^ cells were transfected with miR-1224-overexpression. After five days of the above injection, tumor growth was examined every five days. Our group slaughtered the mice on the thirtieth day and then extracted samples from two groups. The width and length of the tumor were measured at the indicated time, and the bodyweight of mice was recorded after the samples were collected. The animal-related protocol was approved by the Animal Ethics Committee of The Affiliated Huaian No.1 People’s Hospital of Nanjing Medical University.

### Bioinformatics Analysis

The gene ontology (GO) analyses (including BP: biological process, CC: cellular components and MF: molecular functions) and biological pathways analyses were conducted with FunRich software(http://www.funrich.org/). The interaction network of DE miRNAs-potential target genes was generated by the miRNet algorithm (33). The potential miRNAs target genes were predicted by starbase, TargetScan, and miRDB online algorithms (34–36). The potential transcription factors (TFs) for miR-1224 were predicted through FunRich software(http://www.funrich.org/). Additionally, the potential-TFs-miR-1224 interaction network was generated by Cytoscape software(https://cytoscape.org/).

### Statistical Analysis

All the statistical analysis was conducted using SPSS19.0 for Windows (SPSS, Inc., Chicago, IL, USA). The experiment was repeated three times, and each experiment contained three replicates. Furthermore, a student’s t-test was performed to analyze the differences between the two groups. Data from more than two groups were compared using one-way ANOVA. Kaplan-Meier methods were employed for the overall survival curve, and the differences were tested by the log-rank test. Besides, survival data were assessed using a Cox proportional hazards model. *p* < 0.05 indicated statistical significance.

## Results

### Down-Regulation of miR-1224 in Both GC Tissues and Cell Lines

**Figure 1:**
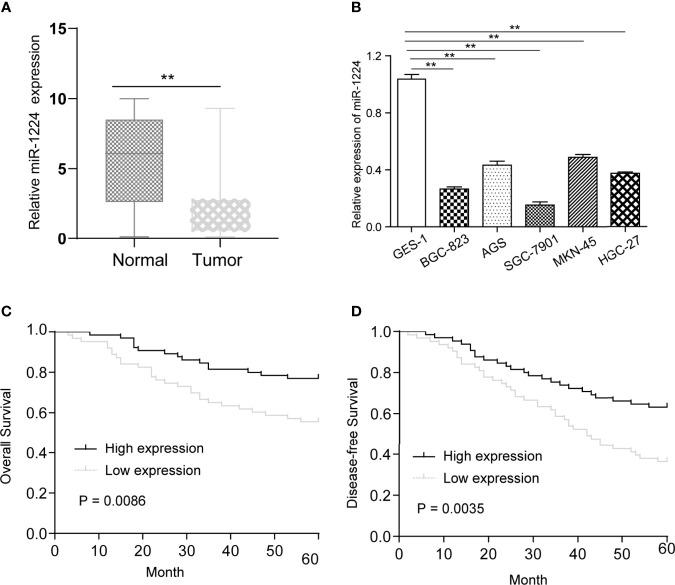
MiR-1224 was down-regulated in GC. **(A)** The qPCR results of miR-1224 in 128 pairs of GC tissues and adjacent tissues. **(B)** The qPCR analysis of miR-1224 expressions in GC cell lines. **(C)** The overall survivals of GC patients who had higher or lower miR-1224 expression were analyzed by Kaplan-Meier assays and log-rank test. **(D)** Disease-free survivals. **p < 0.01. Experiments were repeated three times with three replicates.

RT-PCR was performed to explore whether miR-1224 was dysregulated in GC. It was observed that miR-1224 expression in GC specimens dramatically decreased compared with matched normal specimens (**Figure 1A**). Besides, the decreased expressions of miR-1224 were detected in five GC cells (**Figure 1B**). These findings verified that miR-1224 expressions were reduced in GC tumors.

### Association Between miR-1224 Expression and Patients’ Survival

Then, whether miR-1224 was associated with the clinical progression of GC patients was further explored. Based on the relative miR-1224 expressions in 128 samples, 128 patients were classified into two groups: high-group (n = 63) and low-group (n = 65). The associations between miR-1224 expressions and clinicopathological parameters in GC were summarized in **Table 2**. It can be found that low miR-1224 expression was significantly associated with the TNM stage (*p* = 0.007). Moreover, the Kaplan-Meier survival assay suggested that the low expressions of miR-1224 were positively correlated with poorer overall survival (*p* = 0.0086) and disease-free survival (*p* = 0.0035) of GC patients (**Figures 1C, D**). Finally, the independence of miR−1224 was assessed using multivariate analyses. miR−1224 was a potential independent prognosis factor in GC (*p* < 0.05, **Table 3**). Overall, miR−1224 levels were associated with the clinical progress of GC patients and would be a tumor suppresser and new biomarker.

**Table 2:** miR-1224 expression and clinicopathologic features in GC patients.

Variable	Number	miR-1224 expression		p-value
		High	Low	
Age				NS
<60	66	30	36	
≥60	62	33	29	
Gender				NS
Male	84	41	43	
Female	44	22	22	
Histology/differentiation				NS
Well + moderate	81	43	38	
Poor	47	20	27	
TNM stage				0.007
I + II	94	53	41	
III + IV	34	10	24	

Ns, no significance.

**Table 3:** Multivariate assays of overall survival and disease-free survival in GC patients.

Variable	Univariate analysis			Multivariate analysis		
	RR	95% CI	*p*	RR	95% CI	*p*
Age	1.547	0.667-2.145	0.342	1.323	0.549-1.789	0.234
Gender	1.226	0.538-1.899	0.461	1.078	0.678-1.691	0.431
Histology/differentiation	1.642	0.831-2.334	0.138	1.342	0.652-1.876	0.103
TNM stage	3.672	1.564-5.213	0.001	3.148	1.217-4.778	0.005
miR-1224 expression	3.451	1.437-4.776	0.003	3.063	1.128-4.223	0.008

### Overexpression of miR-1224 Could Suppress the Proliferation of GC Cells

**Figure 2:**
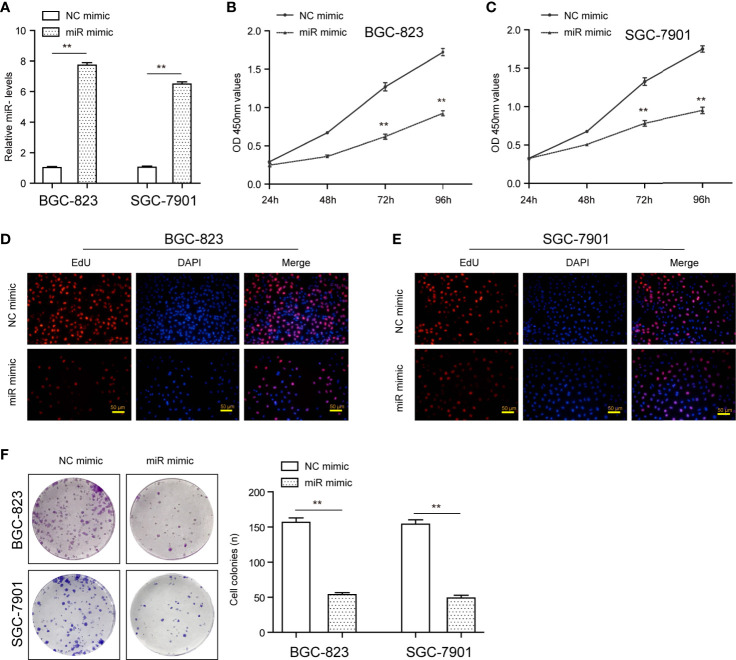
MiR-1224 affected the proliferation of SGC-7901 and BGC-823 cells. **(A)** The qPCR assays determined the expression of miR-1224 in GC cells transfected with miR-1224 mimics. **(B, C)** The cellular growth of miR-1224 mimics-transfected GC cells was detected by CCK-8 assays. **(D, E)** EdU assays evaluated the cell proliferation of GC cells after treatment with miR-1224 mimics. The red color represented proliferative cells. The blue color indicated the nuclei stained by DAPI. **(F)** The colony formation abilities of GC cells were assessed using the colony formation assays. **p < 0.01. Experiments were repeated three times with three replicates.

**Figure 3:**
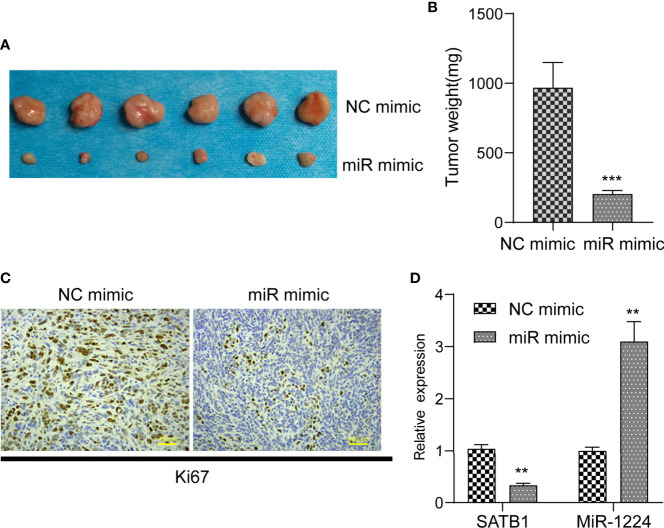
MiR-1224 suppressed tumor growth of GC cells *in vivo*. **(A, B)** The analysis of tumor growth of miR-1224-overexpressed SGC7901 cells in the nude mice. **(C)** HE staining and IHC staining of Ki-67 in two groups. **(D)** RT-PCR assay revealed the expression of miR-1224 and STAT1 in tissues obtained from two groups. **p < 0.01, ***p < 0.001. Experiments were repeated three times with three replicates.

The remarkable reduction of miR-1224 expression in GC implied possible biological function in tumorigenesis. Because the expression of miR-1224 was relatively higher in SGC-7901 and BGC-823, we chose them for further functional assays. Hence, two GC cell lines (SGC-7901 and BGC-823) were transfected with miR-1224 mimics or NC mimics to determine the effects of miR-1224 on cellular growths. Subsequently, qPCR assays were conducted to determine the miR-1224 expression. The data indicated that the levels of miR-1224 were distinctly overexpressed in the transfection of miR-1224 mimics (**Figure 2A**). According to the CCK-8 assays, enforced expression of miR-1224 notably reduced the cellular growth of GC tumor cells (**Figures 2B, C**). Besides, cell proliferation was evaluated through EdU assays. The results suggested that the enhanced expression of miR-1224 resulted in a remarkably decreased cellular proliferation of GC cancer cells, consistent with the results of CCK-8 assays (**Figures 2D, E**). Moreover, the number of tumor cell colonies with miR-1224 overexpression significantly decreased compared to the controls, as demonstrated by colony formation assays (**Figure 2F**). Then, the possible roles of miR-1224 during the growth of SGC7901 cells *in vivo* were studied. Particularly, the enforced miR-1224 expression distinctly alleviated the tumor size and tumor weight (**Figures 3A, B**). As exhibited in **Figure 3C**, the positivity of Ki-67 was reduced *via* miR-1224 promotion. Furthermore, miR-1224 exhibited a higher level in samples from miR mimic group while SATB1 displayed a decreased level (**Figure 3D**). These data suggested that miR-1224 contributed to the inhibitory effects of GC cellular growth.

### Enforced Expression of miR-1224 Depresses the Metastatic Ability of GC Cells

**Figure 4:**
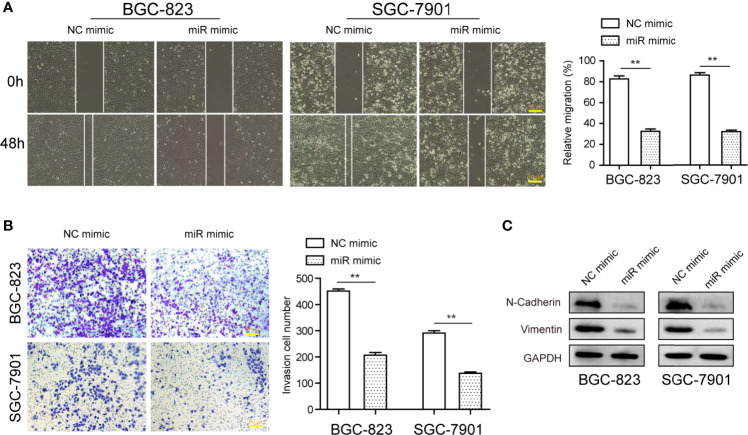
The influence of miR-1224 on the invasion and migration of BGC-823 and SGC-7901 cells. **(A)** Overexpression of miR-1224 reduced the migration of GC cells. **(B)** Ectopic expression of miR-1224 depressed the invasion of GC cells. **(C)** The protein expression of N-cadherin and vimentin was measured with Western blot assays. **p < 0.01. Experiments were repeated three times with three replicates.

Next, the dynamic changes of migratory and invasive ability of tumor cells after the transfection of miR-1224 mimics were monitored to investigate the functional impact of miR-1224 overexpression on GC cell metastatic potentials. Wound healing assays demonstrated that a remarkable reduction in wounded areas was observed in cancer cells transfected with miR-1224 mimics, reflecting that the enhanced expression of miR-1224 markedly abrogated the migration of GC cells (**Figure 4A**). According to the results of transwell assays, the cells transfected with miR-1224 mimics displayed much fewer invasive cells compared with NC mimics-transfected cells. Thus, ectopic expression of miR-1224 contributed to the inhibition of GC cell migration (**Figure 4B**). Considering that epithelial-mesenchymal transition (EMT) was involved in regulating cancer metastasis, western blot assays were performed to detect the levels of EMT-related markers. The marked decline of N-cadherin and vimentin expression was observed in GC cells when they exhibited an overexpressed miR-1224 (**Figure 4C**). These results confirmed that the enhanced expression of miR-1224 attenuated the invasion and migration of GC cells.

### miR-1224 Targets SATB1 3’-UTR and Downregulates Its Expressions in GC Cells

**Figure 5:**
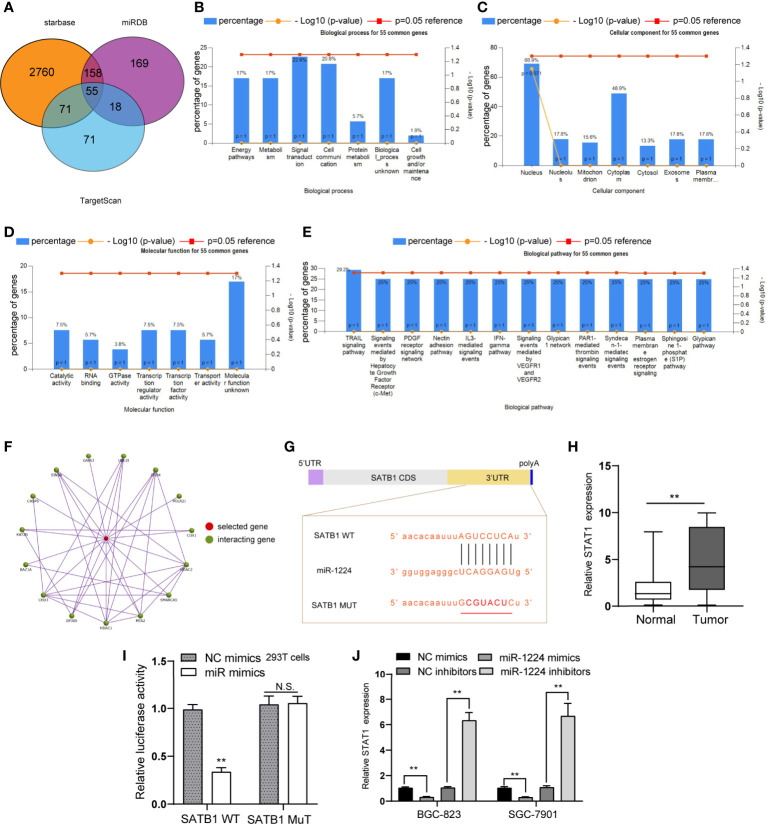
SATB1 is a direct target of miR-1224 in GC cells. **(A)** Venn diagram of the potential target genes of miR-1224. **(B)** Biological processes of the 55 common genes. **(C)** The cellular components analyses. **(D)** The molecular functions analyses for the 55 common genes. **(E)** The biological pathways analyses for the 55 common genes. **(F)** The SATB1-genes interaction network. **(G)** miR-1224 and its binding sequence in the wild-type and mutant 3’UTR of SATB1 mRNA. **(H)** The levels of SATB1 in GC tissues and matched non-tumor samples were determined using qPCR. **(I)** Luciferase activities confirmed that miR-1224 directly targeted SATB1. **(J)** The levels of SATB1 were determined by qPCR after the transfection of miR-1224 mimics or inhibitors. **p < 0.01. Experiments were repeated three times with three replicates. Ns, no significance.

Three online databases (starbase, miRDB, and TargetScan) were explored for their possible targets that displayed oncogenic properties to reveal the mechanisms involved in the tumor-suppressive roles caused by miR-1224 overexpression. The Venn diagram illustrated that there were 55 commonly potential target genes (**Figure 5A**). Next, whether the 55 common genes were associated with cancer development was investigated. Therefore, the gene ontology (GO) analysis was conducted using FunRich software. Biological process analysis revealed that the 55 common genes were correlated with energy pathways, metabolism, and signal transduction (**Figure 5B**). The cellular components and molecular functions for the 55 common genes were also analyzed (**Figures 5C, D**). Besides, biological pathways demonstrated that the 55 common genes were related to multiple cancer-correlated pathways, such as TRAIL signaling, VEGF and VEGFR signaling, S1P pathway (**Figure 5E**). Among the 55 commonly potential target genes, SATB1, a previously reported oncogene, attracted our attention. This was because the SATB1-gene interaction network generated by FunRich software suggested that SATB1 was closely associated with other cancer-correlated genes, such as GATA3, HDAC1, and MTA2 (**Figure 5F**). Afterward, the above prediction was confirmed. The binding site between miR-1224 and the 3’-UTR of SATB1 mRNA was presented in **Figure 5G**. We performed qPCR and found that SATB1 levels were distinctly upregulated in GC tumor specimens compared to normal tissues (**Figure 5H**). Additionally, dual-luciferase reporter assays on 293T cells were conducted to reveal that luciferase activity distinctly decreased in 293T cells after transfection of miR-1224 mimics and the wild-type 3’-UTR of SATB1 reporters (**Figure 5I**). Moreover, overexpression of miR-1224 could suppress the SATB1 levels, and the inhibition of miR-1224 exhibited an opposite function that miR-1224 knockdown led to markedly increased SATB1 expression (**Figure 5J**). These findings verified that miR-1224 could target SATB1 in GC cells.

### The Up-Stream Transcription Factors Analysis for miR-1224

**Figure 6:**
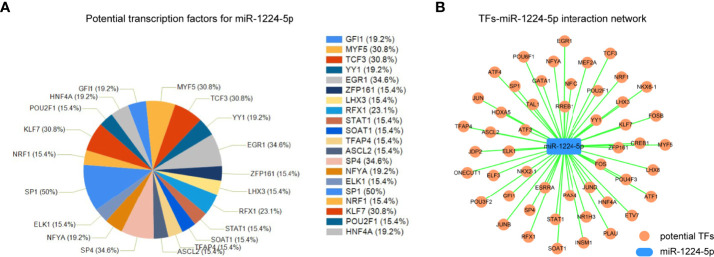
The TFs-miR-1224 interaction network. **(A)** The potential TFs for miR-1224. **(B)** The TFs-miR-1224 interaction network. Experiments were repeated three times with three replicates.

Since the above data validated that miR-1224 was aberrantly downregulated in GC, the mechanisms that contributed to its dysregulation were investigated. Considering that transcription factors (TFs) were the critical regulators for the modulation of miRNAs expression, FunRich software was applied to analyze the potential TFs that could regulate miR-1224 expression. The first twenty potential TFs, including SP1, SP4, TCF3, and KLF7, were presented in **Figure 6A**. Besides, the potential TFs-miR-1224 interaction network was generated using Cytoscape software, as illustrated in **Figure 6B**. Therefore, we uncovered the possible molecular mechanisms leading to miR-1224 dysregulation and the TFs-miR-1224 interaction network.

### Ectopic Expression of miR-1224 Results in the Inhibitory Effects on the Wnt/β-Catenin Pathway

**Figure 7:**
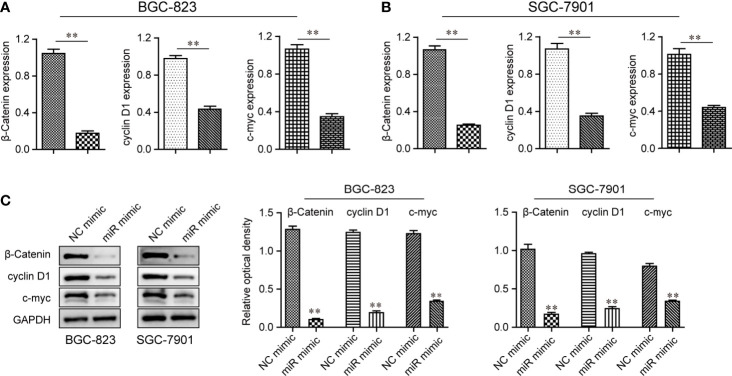
The activity of the Wnt/β-catenin signaling pathway was repressed by up-regulation of miR-1224 in BGC-823 and SGC-7901 cells. **(A, B)** The qPCR assays examined the mRNA levels of β-catenin, cyclin D1, and c-myc in BGC-823 and SGC-7901 cells. **(C)** The protein expressions of c-myc, cyclin D1, and β-catenin in BGC-823 and SGC-7901 cells were reduced when the cells were transfected with miR-1224 mimics. **p < 0.01. Experiments were repeated three times with three replicates.

As suggested by the above findings, miR-1224 served as a tumor suppresser in GC. Next, the detailed mechanisms by which miR-1224 modulated the development and progression of GC were revealed. Since several essential signaling pathways (particularly Wnt/β-catenin signaling) acted as vital players in tumor growth, qPCR and western blot assays were conducted to evaluate the levels of Wnt/β-catenin signaling associated molecules. According to the results of qPCR assays, the mRNA levels of β-catenin and its two targeting genes (cyclin D1 and c-myc) dramatically decreased in GC cells after miR-1224 upregulation (**Figures 7A, B**). As certified by western blot assays, enforced expression of miR-1224 notably repressed the protein expression of c-myc, β-catenin, and cyclin D1 in BGC-823 and SGC-7901 cells. This was consistent with the results of qPCR assays (**Figure 7C**). These results demonstrated that enforced miR-1224 expression suppressed the activity of the Wnt/β-catenin pathway in GC.

## Discussion

GC appears to be malignant due to its aggressive characteristics. In China, the incidence is increasing recent years (37). Regardless of advancements in treatments over the past few decades, the prognosis remains extremely poor for most GC patients. Prediction of the prognosis of GC patients is imperative for clinical management, and more and more cancer biomarkers have been identified in various tumors (38). Unfortunately, the clinical use of biomarkers in the management of patients with GC lags behind other solid tumors. Recently, miRNAs became novel candidates for diagnosis and prognosis of various tumors (including GC) ascribed to its frequent dysregulated expression and the critical effects in regulating the progression of GC (39, 40). This study focused on a novel miRNA miR-1224.

In this study, the levels of miR-1224 in both GC were examined using RT-PCR. The results confirmed that miR-1224 was lowly expressed in both GC tissues and cell lines, consistent with the results of online data. Subsequently, clinical assays indicated that low miR-1224 expression was associated with advanced tumor stages, suggesting that miR-1224 may predict an unfavorable outcome of GC patients. Thus, whether miR-1224 expression influence the long-term survival rate of GC patients was confirmed by collecting five-year follow-up data and performing Kaplan-Meier assays. It was revealed that patients with low expressions of miR-1224 had shorter overall survivals. Moreover, Cox regression analysis demonstrated that low miR-1224 expression independently predicted a worse prognosis. Hence, miR-1224 could be used as a potential biomarker for GC patients. To sum up, miR-1224, together with other functional miRNAs, could be used as potential novel cancer biomarkers for the diagnosis and prognosis of GC patients.

More and more miRNAs have been reported to act as tumor promotors or anti-oncogenes in various tumors, including GC. For instance, overexpression of miR-616-3p promoted metastasis of GC by modulating EMT progress (41). MiR-491-5p inhibited GC metastasis by regulating SNAIL and FGFR4 (42). Wu et al. reported that overexpression of miR-218 reduced tumor behaviors of human GC cells by regulating the Bmi-1/Akt signaling pathway (43). These findings highlighted the clinical application of miRNAs as novel therapeutic targets for GC patients. Recently, miR-1224 was reported to be lowly expressed in glioma, reflecting a poor survival in glioma patients. Their gain-of-function assays confirmed that up-regulation of miR-1224 displayed increased abilities of the invasion and growth of glioma cells by targeting CREB1 However, whether miR-1224 may display a similar in other tumors remains unclear. The data obtained in this study expanded the roles of miR-1224 in GC. Our experiments demonstrated that overexpression of miR-1224 distinctly suppressed the proliferation and metastasis of GC cells. Moreover, the results of Western blot indicated that up-regulation of miR-1224 inhibited the progress of the EMT pathway. These data revealed that miR-1224 may function as a tumor suppressor in GC. Then, the potential mechanism involved in the effects of miR-1224 in GC cells was further explored through bioinformatics analysis with the potential targeting genes of miR-1224. The results suggested that the 55 common genes were associated with cancer development. Besides, we predicted that SATB1 may be a potential target of miR-1224. According to the data of luciferase reporter assays and RT-PCR, SATB1 was identified as a target gene of miR-1224. Previously, the effects of SATB1 acting as a tumor promoter had been demonstrated. It can be concluded that miR-1224 acted as a tumor suppressor by targeting SATB1.

The Wnt signaling pathway is a well-studied evolutionarily conserved pathway that has been demonstrated to be involved in the modulation of various cellular processes, such as cellular proliferation, differentiation, and apoptosis (44). Additionally, Aberrant Wnt/β-catenin signaling is widely implicated in various cancers and other disease states (45). There is little research on the upstream regulators of the Wnt/β-catenin pathway in GC. Recent evidence suggested that miRNAs eventually display their functional roles in cancer through the regulation of several signaling pathways, such as AMPK/mTOR, NF-κB, and Wnt/β-catenin pathway (46–48). In this study, the results of Western blot illustrated that overexpression of miR-1224 distinctly inhibited the expressions of β-catenin, cyclin D1, and c-myc at both mRNA and proteins levels, indicating that the Wnt/β-catenin pathway was inactivated. Thus, miR-1224 functioned as a tumor suppressor by inhibiting the Wnt/β-catenin pathway.

Our study had several limitations: firstly, the potential mechanisms involved in miR-1224 dysregulation was not explored. The small sample size of the present study was also a limitation. In addition, we did not unveil the downstream molecules which SATB1 regulated.

## Conclusions

The conclusions of this research are drawn as follows. First, decreased miR-1224 expression is a common event and may be used as an independent marker for the prognosis prediction in GC. Furthermore, miR-1224 could suppress the proliferation and metastasis of GC cells through the modulation of SATB1. Therefore, the understanding of miR-1224’s precise role in the pathogenesis of GC can contribute to the development of a novel biomarker and therapeutic strategy for GC.

## Data Availability Statement

The raw data supporting the conclusions of this article will be made available by the authors, without undue reservation.

## Ethics Statement

The studies involving human participants were reviewed and approved by The Affiliated Huaian No.1 People’s Hospital of Nanjing Medical University. The patients/participants provided their written informed consent to participate in this study. The animal study was reviewed and approved by The Affiliated Huaian No.1 People’s Hospital of Nanjing Medical University.

## Author Contributions

Conception and design: G-DH and JZ. Acquisition of data: G-DH, YS, and H-XH. Analysis and interpretation of data: M-YT and Y-QL. Writing, review, and/or revision of the manuscript: G-DH, H-XH, and JZ. All authors contributed to the article and approved the submitted version.

## Funding

This study was supported by grants from The Development on Science and Technology Foundation of Nanjing Medical University (No. NMUB2019355).

## Conflict of Interest

The authors declare that the research was conducted in the absence of any commercial or financial relationships that could be construed as a potential conflict of interest.

## Publisher’s Note

All claims expressed in this article are solely those of the authors and do not necessarily represent those of their affiliated organizations, or those of the publisher, the editors and the reviewers. Any product that may be evaluated in this article, or claim that may be made by its manufacturer, is not guaranteed or endorsed by the publisher.
